# Research progress on the correlation between islet amyloid peptides and type 2 diabetes mellitus

**DOI:** 10.1515/med-2024-1124

**Published:** 2025-03-17

**Authors:** GuangZhi Li, Dongmei Zhang

**Affiliations:** Department of Basic Medical, Jiangsu College of Nursing, JiangSu, 223005, China

**Keywords:** islet amyloid peptides, type 2 diabetes mellitus, human

## Abstract

**Background:**

Type 2 diabetes mellitus (T2DM) is a chronic metabolic disorder characterized by insulin resistance and β-cell dysfunction. A hallmark of T2DM pathology is the accumulation of toxic amyloid polypeptides in and around pancreatic islet cells, leading to the progressive loss of β-cell populations. Human islet amyloid polypeptide (hIAPP), also known as amylin, is a 37-amino acid peptide hormone primarily produced by pancreatic β-cells. hIAPP aggregation and amyloid formation are strongly correlated with β-cell death and disease severity in T2DM patients.

**Objectives:**

This article aims to review the current research progress on the correlation between hIAPP and T2DM, focusing on the molecular mechanisms and potential therapeutic strategies.

**Methods:**

We conducted a comprehensive literature review covering recent studies on the molecular structure, physiological function, and pathological mechanisms of hIAPP. Key areas include biosynthesis, monomer structure, and the formation of hIAPP fiber structures. Additionally, we examined the mechanisms of hIAPP-induced β-cell death, including oxidative stress (OS), endoplasmic reticulum stress (ERS), impaired cell membrane and mitochondrial functions, and inflammatory factors.

**Results:**

Our review highlights the critical role of hIAPP in the pathogenesis of T2DM. Specifically, we found that hIAPP biosynthesis and monomer structure contribute to its physiological functions, while hIAPP aggregation forms toxic amyloid fibers, contributing to β-cell dysfunction. OS, ERS, impaired cell membrane and mitochondrial functions, and inflammatory factors play significant roles in hIAPP-induced β-cell death. There is a strong correlation between hIAPP aggregation and the severity of T2DM, and potential therapeutic approaches using small molecule inhibitors to prevent hIAPP aggregation and fibrosis are discussed.

**Conclusion:**

Understanding the molecular mechanisms of hIAPP in T2DM provides insights into potential therapeutic targets and preventive strategies. Future research should focus on developing more effective treatments targeting hIAPP aggregation and its downstream effects.

## 1 Introduction

Type 2 diabetes mellitus (T2DM) is a chronic metabolic disorder characterized by insulin (Ins) resistance and β-cell dysfunction [1]. A hallmark of T2DM pathology is the accumulation of toxic amyloid polypeptides in and around pancreatic islet cells, leading to the progressive loss of β-cell populations. Human islet amyloid polypeptide (hIAPP), also known as amylin, is a 37-amino acid peptide hormone primarily produced by pancreatic β-cells. hIAPP aggregation and amyloid formation are strongly correlated with β-cell death and disease severity in T2DM patients [2]. This article reviews the research progress on the correlation between hIAPP and T2DM, focusing on the molecular mechanisms and potential therapeutic strategies. The main feature of diabetes is abnormally high blood sugar levels. Diabetes has become one of the most widespread major diseases globally, affecting over 170 million people [1,2]. Globally, this number is projected to grow rapidly in developing countries in Africa, Asia, and South America, with an estimated increase of nearly 50% by 2023. The prevalence of diabetes in more developed societies has reached about 6%, and an even more alarming 4% of obese white adolescents have diabetes and 25% have abnormal blood sugar regulation [3,4]. Diabetes can lead to many serious complications, including blindness, kidney failure, and cardiovascular disease. Due to its high incidence and clinical complexity, diabetes, as a chronic disease, poses a significant medical and socioeconomic challenge to human society [4].

There are two main types of diabetes. Type 1 diabetes mellitus refers to Ins-dependent diabetes mellitus, where Ins production and beta-cell secretion in the islets are disrupted by the body’s autoimmune response, resulting in Ins deficiency at a young age. Type 2 diabetes (T2D), or non-Ins-dependent diabetes mellitus [5,6], involves initial defects primarily characterized by Ins resistance. In other words, Ins target cells, especially muscle and liver cells, do not respond normally to Ins, even when compensated with high levels of Ins. In the early stages of T2D, basal blood sugar levels may remain normal due to increased Ins production and secretion (hyperinsulinemia) [6,7]. Studies have found that more than 90% of patients with T2D have islet amyloidosis, which is more common than diabetes syndrome [8]. The extent of amyloid invasion and the number of affected islets determine the severity of diabetes. In contrast, only a very small number of islets are involved in healthy individuals, and while amyloid can be observed in the islets of the elderly, it is present in smaller quantities compared to T2D patients. Therefore, the vast majority of people with T2D have islet amyloid deposits, and amyloidosis is a hallmark of T2D [9].

**Figure 1:**
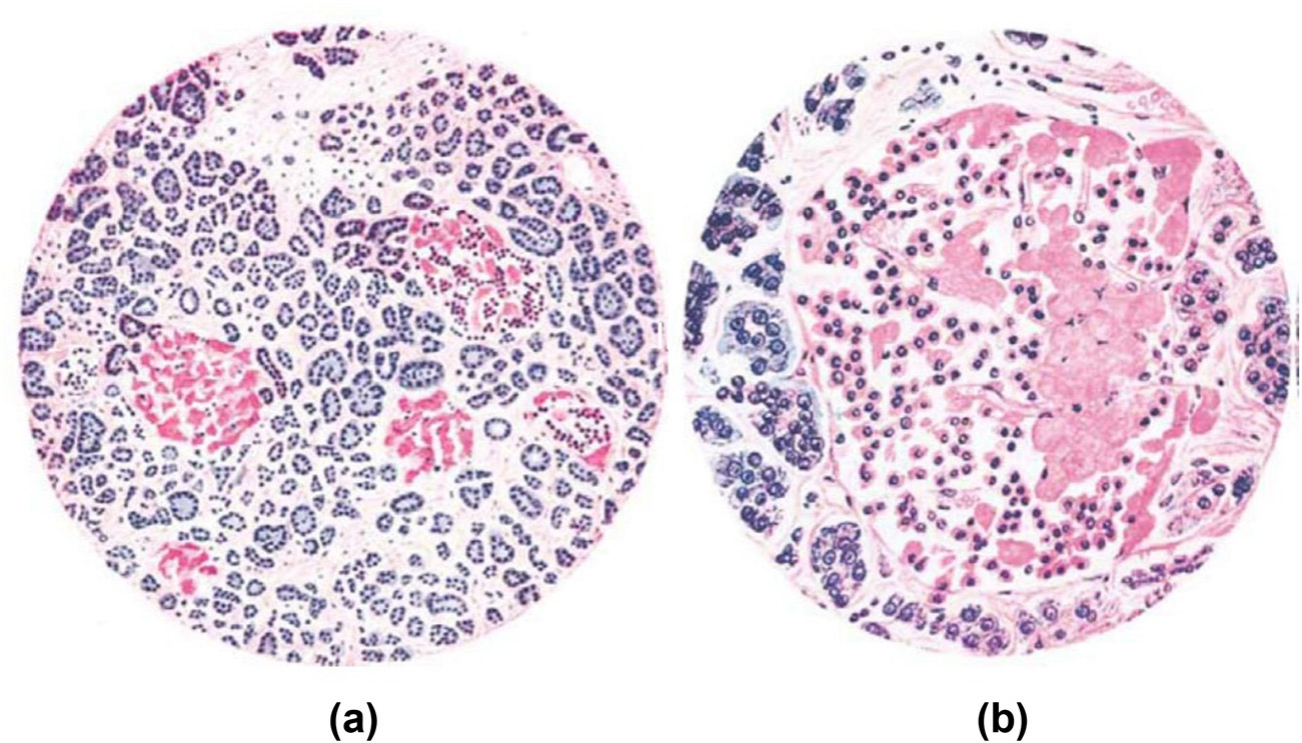
Micrograph of islets replaced by amyloid precipitate (a) and micrograph of individual islets (b).

In T2D, amyloid is localized and deposited in islets (Figure 1). Congo red and thioflavin S staining show a green birefringent light under a polarized light microscope. Electron microscopy reveals that the amyloid deposits consist of fibers of varying lengths with an average diameter of 7–10 nm [10]. The amyloid is composed of islet amyloid polypeptide (IAPP), apolipoprotein E (ApoE), and heparan sulfate perlecan. IAPP is unique to islet amyloid, while ApoE and heparan sulfate perlecan are also found in other amyloidosis diseases and are common components of amyloid [11–13].

**Figure 2:**
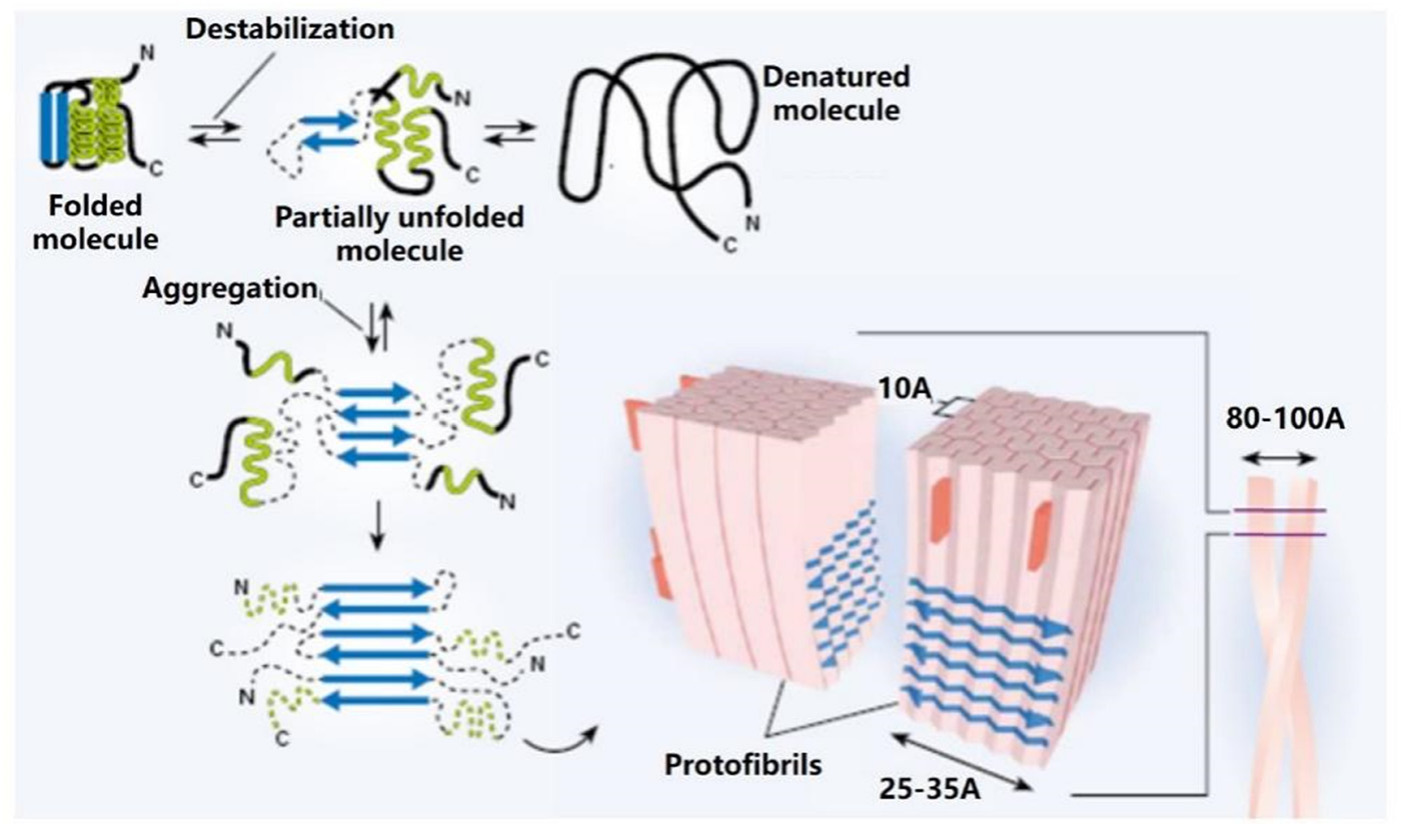
Aggregation of amyloid polypeptides in human islets.

Amyloid deposits have a typical ultrastructure called filament: due to hydrogen bonding between regions of the protein molecule rich in a “beta-folded” structure, proteins stack on each other to form filament further amyloid deposits (Figure 2) [14,15].

hIAPP-derived aggregates or amyloid deposition have toxic effects on islet beta cells and are the main cause of T2DM. hIAPP, also known as amylin, is stored in islet beta cells along with Ins [15,16]. hIAPP deposition is present in 95% of T2DM patients, which is positively correlated with disease severity and reduces islet beta cell function. This article reviews the research progress on the correlation between hIAPP and T2DM [16].

## 2 Molecular structure and physiological function of hIAPP

### 2.1 Biosynthesis and monomer structure of hIAPP

**Figure 3:**
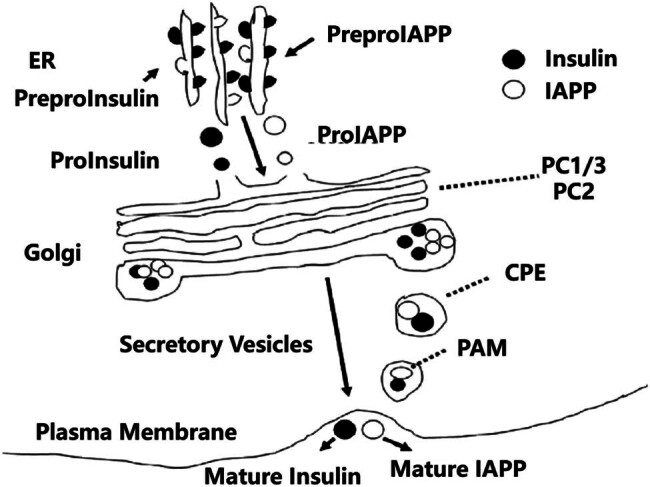
Biosynthesis of IAPP in β cells.

The hIAPP gene is located on the short arm of chromosome 12, and IAPP is also included as a member of the calcitonin family due to its high sequence homology (46%) with calcitonin and calcitonin gene-related peptides, as well as the same signaling pathway as other members of the calcitonin family [17]. In the β cell, the IAPP precursor, PreproIAPP, containing 89 residues, is first synthesized. Then, PreproIAPP was hydrolyzed by signal peptidase in the endoplasmic reticulum (ER) to form ProIAPP containing 67 residues. The latter was digested by prohormone converting enzymes PC1/3 and PC2 into secretory vesicles in Golgi and then carboxypeptidase E (CPE). Further action of CPE and peptidylamdating monooxygenase (PAM) resulted in a mature IAPP composed of 37 amino acids (Figure 3) [18,19]. It is worth noting that the biosynthesis and secretion pathway of Ins is highly consistent with that of IAPP, and it is also formed by its precursor preproinsulin through the action of PC1/3, PC2, and CPE in turn. After undergoing the same biosynthesis and processing steps, Ins and IAPP are co-stored in secretory vesicles and secreted together with IAPP after stimulation, the only difference is that the synthesis of Ins does not require the action of PAM [19–21].

A large number of studies have shown that hIAPP monomer mainly exists in the form of random coil under physiological conditions, but sequence software analysis has found that some sequence fragments of hIAPP tend to form local secondary structures [21,22]. Experimental studies also found that the structure of the hIAPP monomer would change significantly under some solvent conditions. For example, some scholars applied circular dichroism and fluorescence studies and found that a large number of A-helix structures would be formed in trifluoroethanol under the hIAPP monomer [22,23].

### 2.2 Physiological function of hIAPP

hIAPP plays an important role in metabolism, especially blood sugar control. hIAPP inhibits food absorption and gastric emptying. In the same environment, the hIAPP knockout mice had accelerated gastric emptying and were 30% heavier than normal mice. Accelerated gastric emptying was present in patients with type 1 diabetes and T2D, but the rate of gastric emptying decreased significantly after IAPP injection [24,25].

hIAPP also inhibits Ins secretion. The study found that the cholinergic nervous system is involved in the regulation of Ins secretion by beta cells, and hIAPP can inhibit Ins secretion by blocking the cholinergic mechanism [26]. hIAPP is also able to inhibit the secretion of Ins caused by glucose stimulation, but only when IAPP concentrations are greater than 10 mmol/L. Given the low concentration of hIAPP circulating under normal physiological conditions (pmol/L level), this mechanism may not work under normal conditions [27,28].

hIAPP also has a certain inhibitory effect on glucagon secretion. Studies have found that IAPP can inhibit glucagon secretion by changing the plasma glucose concentration in the blood and inhibiting Ins secretion. However, hIAPP does not affect the normal function of glucagon, and glucagon can still play a normal role in hypoglycemia [28,29].

In people with type 1 diabetes, the total amount of hIAPP and Ins secreted is insufficient due to the destruction of islet beta cells, leading to the development of diabetes symptoms [29]. Clinical studies have shown that increasing hIAPP levels in patients with type 1 diabetes have a good effect on weight control, postprandial blood glucose control, and long-term blood glucose control. Based on these studies, the American company Amylin developed the world’s first HiAPP-based analogue drug – Symlin (pramlintide acetate), which is currently mainly used as an Ins adjuvant in patients with type 1 diabetes [30,31].

In addition to regulating blood sugar, hIAPP also has some other physiological functions, such as dilating blood vessels and lowering blood calcium; activate the renin-angiotensin system; inhibit gastric acid secretion and keep gastric mucosa intact; inhibition of atrial natriuretic peptide secretion; help amino acids cross the blood–brain barrier, raise body temperature, etc. [31,32].

## 3 Structure formation of hIAPP fiber

hIAPP self-assembles amyloid fibers at micromolar concentrations through a classical nucleation growth mechanism that exhibits S-shaped aggregation dynamics characterized by lag, growth, and plateau periods. hIAPP nucleation occurs in the late stage of the assembly of soluble hIAPP monomers into oligomers, which is largely affected by temperature, peptide concentration, ionic strength, and pH value [33]. These nuclei further develop and elongate, forming fibrils during the growth phase. The fibril produces more fibril through secondary nucleation to catalyze new fibril and finally reaches homeostasis. The dynamic aggregation process of hIAPP involves the formation of a large number of different, transient oligomer intermediates and has a potential role in cytotoxicity and fibril formation [33,34].

## 4 Mechanism of hIAPP-induced death of pancreatic β cells

The fibril toxicity of hIAPP directly damages the islet beta cells, and the oligomer intermediates also damage the islet beta cells. The mechanisms of injury include oxidative stress (OS), ER stress (ERS), mitochondrial dysfunction, cell membrane rupture, islet inflammation, and injury, which aggravate the toxicity of islet beta cells [34,35].

### 4.1 OS

OS is associated with increased reactive oxygen species (ROS) in islet beta cells [36]. ROS are highly reactive molecules produced as a natural byproduct of normal metabolism and cellular processes. In healthy conditions, the body maintains a balance between ROS production and antioxidant defenses [37]. However, in pathological states such as T2DM, this balance is disrupted, leading to OS. The role of ROS in β-cell function is highly dependent on the timing and intensity of the signals [38]. β-cells are particularly susceptible to OS compared to other cell types because they have a limited capacity to scavenge oxidants [39]. Transient and moderate production of mitochondrial ROS is crucial for promoting β-cell function and glucose-stimulated Ins secretion, mimicking the effects of glucose [40]. However, chronic and sustained elevation of ROS due to inflammation or excessive glucose and fatty acid concentrations impairs β-cell function by inhibiting ROS signaling and inducing mitochondrial damage, which in turn leads to increased ROS production.

Increased mitochondrial superoxide production is activated by the peroxidation of mitochondrial phospholipids [6]. Morphological studies have shown that the number of β-cell mitochondria is similar in β-cells from patients with T2D and from non-diabetic donors [41]. However, diabetic pancreatic islets exhibit a significantly higher volume of mitochondrial density, which is associated with increased ROS production. Consistent with this observation, UCP2 protein levels are upregulated in pancreatic islets from T2D patients compared to non-diabetic patients [42].

Glucose is the main energy source for the electron transport chain in islet beta cells. In T2DM patients, elevated blood glucose levels lead to an increased flux of glucose through the electron transport chain, resulting in higher levels of ROS [43]. This excessive ROS production can damage cellular components, including proteins, lipids, and DNA, ultimately contributing to beta-cell dysfunction and impaired Ins secretion [44]. Glucose is the main energy source of the electron transport chain, and T2DM patients increase their energy source due to high blood sugar, resulting in higher levels of ROS [36]. Like other amyloid proteins, the formation and decomposition of hIAPP amyloid proteins are related to ROS production. The formation of hIAPP fibers and oligomers leads to the appearance of OS in pancreatic tissue and the loss of β cell mass in pancreatic islets. Inhibiting amyloid aggregation can reduce OS.

Furthermore, ROS can oxidize cardiolipin (CL) and other mitochondrial inner membrane phospholipids, leading to the permeabilization of the outer mitochondrial membrane and the subsequent release of cytochrome c into the cytosol. This process initiates apoptosis, ultimately resulting in a reduction of β-cell mass. The oxidation of CL disrupts the integrity of the mitochondrial membrane, causing the release of pro-apoptotic factors such as cytochrome c. These factors then activate caspase cascades, leading to the execution phase of apoptosis. As a result, β-cell mass is significantly reduced, contributing to impaired Ins secretion and the progression of T2D.

### 4.2 ERS

The ER in β cells has a high capacity for protein synthesis and folding [45]. However, during Ins resistance, β cells must synthesize Ins beyond their normal folding and secretion capacity, thereby activating the unfolded protein response (UPR). The UPR is an adaptive signaling pathway designed to promote cellular survival when misfolded proteins accumulate in the ER. The UPR signaling sensors include inositol-requiring enzyme 1, PKR-like ER kinase, and activating transcription factor 6. If the UPR is chronically activated and the protein-folding demand in the ER exceeds its capacity, unfolded proteins begin to accumulate, leading to ER stress and ultimately cell death [46].

It has been reported that defects in ERS, ER-associated protein degradation, and the UPR can all contribute to β-cell death through the aggregation of hIAPP [47]. In cases where intracellular aggregates generate toxicity, proIAPP and partially processed proIAPP may be among the harmful species, as deficient processing of proIAPP has been demonstrated in diabetes [48]. Post-translational modifications are completed in the Golgi apparatus and Ins secretory granules [49]. The ERS signaling pathway activates protein kinase, transcription factor CHOP, etc., causing the UPR, which increases the intensity of ERS and decreases protein synthesis. If the UPR is unable to cope with the increase of hIAPP, the accumulation of hIAPP in cells can activate the apoptotic pathway, leading to the damage and death of islet beta cells.

### 4.3 Cell membrane and mitochondrial function are impaired

Mechanistic studies of IAPP-induced membrane disruption remain an active area of research, with various models being proposed. While some studies support a detergent or carpeting mechanism, others suggest a pore-like mechanism [50]. The toxic effect of hIAPP on islet beta cells was related to its ability to penetrate the cell membrane. Compared to the mature hIAPP fibrous structure, the oligomer intermediates have a higher membrane destruction capacity, and the hIAPP oligomer inserts into the membrane bilayer hydrophobic region, resulting in membrane rupture before fibril formation [51]. Therefore, hIAPP oligomers with high membrane permeability and low solubility can destroy the lipid accumulation on the membrane surface, increase the double-layer conductivity, thin and fracture the membrane, and then collapse the phospholipid bilayer [52]. In the islets of hIAPP transgenic mice and T2DM patients, cytoplasmic hIAPP oligomers were found near morphologically damaged mitochondria and ruptured mitochondrial membranes. The toxic effect of hIAPP on rat insulinoma beta cells can lead to mitochondrial rupture, mitochondrial membrane potential loss, and ATP depletion [53,54].

### 4.4 Inflammatory factor

Inflammatory stress plays a crucial role in the pathogenesis of T2DM. Indeed, mild inflammation within the pancreatic islets can be detected in patients with T2DM [55]. The use of anti-inflammatory drugs has been shown to produce a slight decrease in blood glucose levels, suggesting the involvement of inflammation in the development of T2DM. However, it remains unclear whether these anti-inflammatory drugs act directly on the pancreatic islets or through their effects on other relevant metabolic tissues.

This inflammation can arise from various sources, including nutritional overload, proinflammatory cytokines, pancreatic amylin accumulation, and ERS [56]. One of the organelles significantly affected by inflammation is the mitochondrion. Mitochondrial dysfunction leads to impaired ATP production and induces pro-apoptotic mechanisms, contributing to β-cell failure. Mitochondrial autophagy, also known as mitophagy, is an important mechanism that helps counteract the accumulation of damaged mitochondria following inflammatory processes. This process utilizes the autophagic mechanism to specifically remove dysfunctional mitochondria, thus maintaining cellular homeostasis. Any disruption in mitochondrial autophagy sensitizes pancreatic cells to inflammation, making them more vulnerable to OS and apoptosis [57].

Mitochondrial autophagy serves as a critical survival mechanism for pancreatic β-cells in response to inflammation. OS, characterized by an imbalance between ROS and antioxidant defenses, exacerbates mitochondrial dysfunction [58]. This leads to increased ROS production, further damaging mitochondrial integrity and ultimately resulting in β-cell failure. The accumulation of ROS due to impaired antioxidant defenses contributes to mitochondrial dysfunction and the progressive decline of pancreatic β-cell function. IL‑1β is an inflammatory cytokine associated with T2DM and is associated with cytotoxicity. Increased hIAPP activates inflammatory cells to produce IL‑1β, leading to islet beta cell death. Islet macrophages containing amyloid protein in hIAPP transgenic mice expressed higher levels of inflammatory factors such as IL-1β, IL-1α, and TNF‑α [41]. Studies involving T2DM, T2DM alone, and non-T2DM patients with islet amyloid deposition have shown that amyloid-rich islet beta cells are significantly damaged, and a large number of macrophages are mixed with OS-related DNA damage.

In summary, inflammatory stress and the resulting mitochondrial dysfunction play pivotal roles in the pathogenesis of T2DM. Targeting these pathways, particularly through the enhancement of mitochondrial autophagy and the reduction of OS, offers promising therapeutic strategies for managing and potentially reversing the progression of T2DM.

### 4.5 Block IAPP aggregation

Through the development of small molecule drugs or peptide inhibitors, researchers have identified substances that can bind to IAPP and prevent it from forming toxic aggregates. One notable study demonstrated the efficacy of a human monoclonal antibody that selectively targets IAPP oligomers. This antibody effectively neutralizes the aggregation toxicity of IAPP by preventing extracellular membrane destruction and reducing cell apoptosis. In experimental models, antibody therapy has shown promising results: when applied to male rats and mice with human IAPP gene transfer, as well as in mouse models of T2D transplanted with human islets, the treatment successfully triggered the clearance of IAPP oligomers. This not only protected the beta cells but also improved overall blood glucose control. These findings suggest that targeting IAPP oligomers with specific antibodies can be a viable therapeutic strategy for managing and potentially treating T2D [59].

### 4.6 Dietary patterns

One of the most effective strategies for controlling T2D is to follow a specific dietary pattern. Diets rich in whole grains, vegetables, fruits, nuts, and legumes, along with moderate alcohol consumption and less red/processed meats, refined grains, and sugary beverages, have been shown to be particularly beneficial. These dietary choices often lead to weight loss, which is associated with many positive health outcomes. For example, weight loss can significantly reduce cardiovascular risk and lower the risk of all-cause mortality. By promoting healthier body composition and improved metabolic function, these dietary modifications contribute to better overall health and well-being. The Okinawa-based Nordic diet for at least 3 months is sufficient to reduce circulating IAPP and IpuPO-IGA levels, which may be the main reason for controlling T2D [48].

## 5 Correlation between hIAPP and T2DM

In non-DM patients, hIAPP and Ins are co-produced and secreted by islets in a 1:100 molar ratio. Studies have found that hIAPP exists in the pancreas of patients with T2DM death, suggesting a correlation between hIAPP and T2DM. Amyloid polypeptide is a special protein aggregation that produces the characteristic β-folded fibril, Ins itself can be converted into fibril, and Ins or proinsulin is the origin of hIAPP. Protein molecular sequencing confirmed that hIAPP fibril was composed of unknown islet beta cell products [42,43].

hIAPP fiber is the main component of amyloid fiber deposition in islets and participates in T2DM by inducing islet β cell death. Primate studies support that islet amyloidosis and apoptosis of islet beta cells are key factors in islet dysfunction. Overexpression of IAPP leads to pancreatic amyloid formation and T2DM progression [43]. The formation of hIAPP amyloid deposition is related to the pathological mechanism of hyaline degeneration of islets in T2DM patients. hIAPP has been found in the brain and cerebrospinal fluid of both Alzheimer’s disease and T2DM patients, but it is uncertain whether it comes from the brain or the pancreas. hIAPP aggregation with nerve damage was also observed in Hiapp-expressing rat brains [44]. At present, there is no effective method to prevent hIAPP aggregation and toxicity in the human body. The mechanisms of hIAPP synthesis, secretion, and degradation are particularly important for exploring hIAPP aggregation in islet beta cells [36,44].

## 6 Small molecule inhibitor of hIAPP aggregation and fibrosis

The aggregation potential of hIAPP in islets is related to the clinical characteristics of T2DM patients, and hIAPP aggregation is less in non-DM patients. There may be effective natural inhibitors of hIAPP oligomerization and fibrosis in human islets, and Ins, C‑P, and zinc ions (Zn^2+^) may be important intracellular factors that help prevent hIAPP aggregation, and the B chain of Ins is the active component that inhibits hIAPP aggregation [45,46]. The inhibition of amyloid formation by metal ions may be due to the different molecular coordination between hIAPP residues, the HIS-18 imidazole ring in hIAPP is the most significant, which reduces the assembly rate of amyloid protein, and the binding of Zn^2+^ with His18 induces the local destruction of hIAPP secondary structure. C‑P, Zn^2+^, and hIAPP form a stable soluble metal–peptide complex to prevent oligomerization and fibrosis, and Zn^2+^ is also a cofactor for Ins to inhibit hIAPP aggregation [46]. Copper ions reduce the toxic effects of hIAPP by preventing the transition of hIAPP from random curling to β-fold-rich oligomers and aggregates to avoid HiAPP aggregation in islet beta cells. Low molecular weight metal-HIAPP complexes are not cytotoxic, and transition metals or chelates may accelerate hIAPP aggregation and toxicity. Many natural products and derivatives, such as small molecule polyphenol compounds isolated from plants, have been shown to inhibit hIAPP aggregation *in vitro* and intracellular [46,47]. It has been found that Tat-CIAPIN1 protein improves the pathological changes of pancreatic beta cells and reduces fasting blood glucose, body weight, and hemoglobin Alc levels in T2DM mouse models. Tat-CIAPIN1 has a protective effect on β-cells and T2DM by inhibiting hIAPP toxicity and regulating MAPK signaling pathway, suggesting that CIAPIN1 may be a therapeutic protein candidate for beneficial regulation of T2DM. On the other hand, biflavonoids are natural compounds with a wide range of biological activities. A study investigating the inhibitory effects of two biflavonoids (arylflavonoids (1) and arylflavonoids (2)) on the formation of hIAPP protofibrils and their interaction mechanisms found that they significantly prevented the self-assembly behavior of hIAPP and disassembled the aged aggregates into small oligomers and monomers. Both compounds showed strong binding affinity for hIAPP mainly through hydrophobic and hydrogen bonding interactions, and the hydroxyl substitution in 1 was superior to the methoxy substitution in 2 for hindering hIAPP aggregation at the same C8 position. 1 and 2 also reduced hIAPP-induced cytotoxicity by decreasing peptide oligomerization.

## 7 Summary and outlook

In summary, hIAPP serves as the primary component of amyloid deposition in islet tissue, assisting Ins in regulating blood glucose levels in non-diabetic individuals and playing a crucial role in the pathogenesis of T2D. Understanding the cytotoxic mechanisms of hIAPP will further elucidate the pathogenesis of T2D. Additionally, as a typical representative of amyloid deposition proteins, studying hIAPP provides valuable insights into the pathogenesis of other amyloid deposition diseases. To expand our understanding of hIAPP, future research should focus on investigating its role and effects in diverse populations, including different ethnicities and age groups. Specifically, studies should explore the genetic and environmental factors that influence hIAPP expression and toxicity across different ethnicities, such as Asian, Caucasian, African, and Hispanic populations, which could reveal unique patterns and potential therapeutic targets. For instance, comparative studies between these ethnic groups might uncover distinct genetic variants that affect hIAPP aggregation and toxicity, providing a basis for ethnicity-specific therapeutic approaches. Additionally, studying hIAPP in various age groups, from pediatric to elderly populations, can help identify age-specific mechanisms and risk factors. Pediatric studies could uncover early biomarkers of hIAPP-related dysfunction, while geriatric studies might reveal the cumulative effects of hIAPP on islet function over time, leading to more targeted interventions and personalized treatment strategies. Given the critical role of hIAPP in T2D, it presents a promising therapeutic target, and future research should focus on developing inhibitors or modulators that can specifically target hIAPP aggregation and toxicity, potentially enhancing the efficacy of existing treatments. By addressing these research prospects, we aim to gain a comprehensive understanding of hIAPP’s role in diverse populations and its potential as a therapeutic target for T2D and related amyloid deposition diseases.
